# Simulating the decentralized processes of the human immune system in a virtual anatomy model

**DOI:** 10.1186/1471-2105-14-S6-S2

**Published:** 2013-04-17

**Authors:** Vladimir Sarpe, Christian Jacob

**Affiliations:** 1 Department of Computer Science, Faculty of Science, University of Calgary, Calgary, Alberta, Canada; 2 Department of Biochemistry & Molecular Biology, Faculty of Medicine, University of Calgary, Calgary, Alberta, Canada

## Abstract

**Background:**

Many physiological processes within the human body can be perceived and modeled as large systems of interacting particles or swarming agents. The complex processes of the human immune system prove to be challenging to capture and illustrate without proper reference to the spacial distribution of immune-related organs and systems. Our work focuses on physical aspects of immune system processes, which we implement through swarms of agents. This is our first prototype for integrating different immune processes into one comprehensive virtual physiology simulation.

**Results:**

Using agent-based methodology and a 3-dimensional modeling and visualization environment (LINDSAY Composer), we present an agent-based simulation of the decentralized processes in the human immune system. The agents in our model - such as immune cells, viruses and cytokines - interact through simulated physics in two different, compartmentalized and decentralized 3-dimensional environments namely, (1) within the tissue and (2) inside a lymph node. While the two environments are separated and perform their computations asynchronously, an abstract form of communication is allowed in order to replicate the exchange, transportation and interaction of immune system agents between these sites. The distribution of simulated processes, that can communicate across multiple, local CPUs or through a network of machines, provides a starting point to build decentralized systems that replicate larger-scale processes within the human body, thus creating integrated simulations with other physiological systems, such as the circulatory, endocrine, or nervous system. Ultimately, this system integration across scales is our goal for the LINDSAY Virtual Human project.

**Conclusions:**

Our current immune system simulations extend our previous work on agent-based simulations by introducing advanced visualizations within the context of a virtual human anatomy model. We also demonstrate how to distribute a collection of connected simulations over a network of computers. As a future endeavour, we plan to use parameter tuning techniques on our model to further enhance its biological credibility. We consider these *in silico *experiments and their associated modeling and optimization techniques as essential components in further enhancing our capabilities of simulating a whole-body, decentralized immune system, to be used both for medical education and research as well as for virtual studies in immunoinformatics.

## Background

Recent years have witnessed a growing interest in systems biology [1-7]. Not only are biological systems themselves better understood, but increased computational power, visualization environments and more readily available distributed computing enhance the value of modeling and simulation. In the literature so far, there has been little concern regarding more sophisticated visualizations in scientific modeling. Noteworthy efforts in this direction include Harvard's BioVisions project [8]. We take the viewpoint, that simulations should involve a high degree of visual realism; visualization then becomes a key part of our modelling approaches. We present our latest 3-dimensional simulations and interactive visualizations of the decentralized processes in the human immune system.

Using agent-based approaches in simulations is another aspect to increase realism in computer simulations. Rules or simple programs and attributes for agents can then drive the overall dynamics of a system of interacting entities, which result in emergent observable patterns [9,10]. An agent-based approach allows simulations to incorporate computational versions of the physical interaction rules that are observed directly in nature. While the agent-based approach does not replace traditional mathematical modeling [11], it rather acts as a strong complement for better understanding complex biological phenomena. Furthermore, coupling agent-based simulations with advanced graphics visualization and intuitive interaction interfaces can appeal greatly to life scientists, who do not have a programming background or any interest in learning new modeling environments. Allowing such biology experts to appreciate the value of computer simulations is key to the advancement and wider acceptance of systems biology [2,7,12]. Finally, making virtual experiments more accessible to biologists, immunologists, and medical researchers will facilitate answers to particularly those research questions not achievable through purely laboratory means.

In this work, we present our latest simulation of the decentralized processes of the human immune system [10,13]. Our simulation consists of different compartmentalized regions -- simulated as agent environments -- communicating with one another to produce high-level emergent effects such as an organism's immunity to harmful pathogens. Each compartment consists of large numbers of agents, with relatively simple behavioural rules, that act collectively in highly sophisticated networks of interactions. We chose the common Influenza A virus infection as the base for our immune system simulation.

### Adaptive immune system

The adaptive immune response results in the elimination of various pathogens such as viruses and other foreign particles. It is also responsible for developing a memory response for future infections with the same antigens. The mechanism through which humans develop immunity to disease-causing pathogens is through the cellular interactions of two different branches of immunity: (1) humoral and (2) cell-mediated. Humoral immunity acts via the production and secretion of antibodies which neutralize harmful antigens. On the other hand, cell-mediated immunity functions via the destruction of infected cells, in order to suppress any further spreading of a virus. Both branches have the ability to create memory cells that can prevent secondary infections with previously encountered viruses [12].

Lymphocytes have a very important role in the adaptive immune system: T cells are responsible for cell-mediated immunity and B cells are responsible for humoral immunity. T cells and B cells are mainly found throughout the different lymphatic organelles, where they are most likely to encounter antigens. Both types of lymphocytes have antigen-specific receptors on their surface that allows them to detect specific antigen molecules. The binding of an antigen to a naive lymphocyte results in the lymphocyte becoming an effector cell, a cell that is directly involved in the immune response.

The infection is initially detected by professional antigen-presenting cells such as macrophages and dendridic cells that are nearby. Upon detection, the antigen-bearing cells migrate to nearby lymph nodes to trigger the immune response. Within the lymph nodes, B cells and T cells interact with the antigen-presenting cells as well as free antigen molecules. Some activated T cells, namely the cytotoxic T cells, travel to the tissue to eliminate infected cells, while a subpopulation, called helper T cells, remain in the lymph node to promote further proliferation of nearby lymphocytes. B cells that develop into plasma cells produce large amounts of antibodies, which travel via the blood stream to the site of infection in order to neutralize virus particles. Neutralization involves the blocking of surface receptors on a virus responsible for entry into a healthy cell. After the infection is successfully defended, some B and T cells remain in the body as long lived memory cells. Upon secondary exposure to the same virus, the memory cells can quickly divide and mobilize to form a swift response, eliminating the virus before any serious effects are noticed by the host organism [14-17].

#### Immune response to influenza A infection

**Figure 1:**
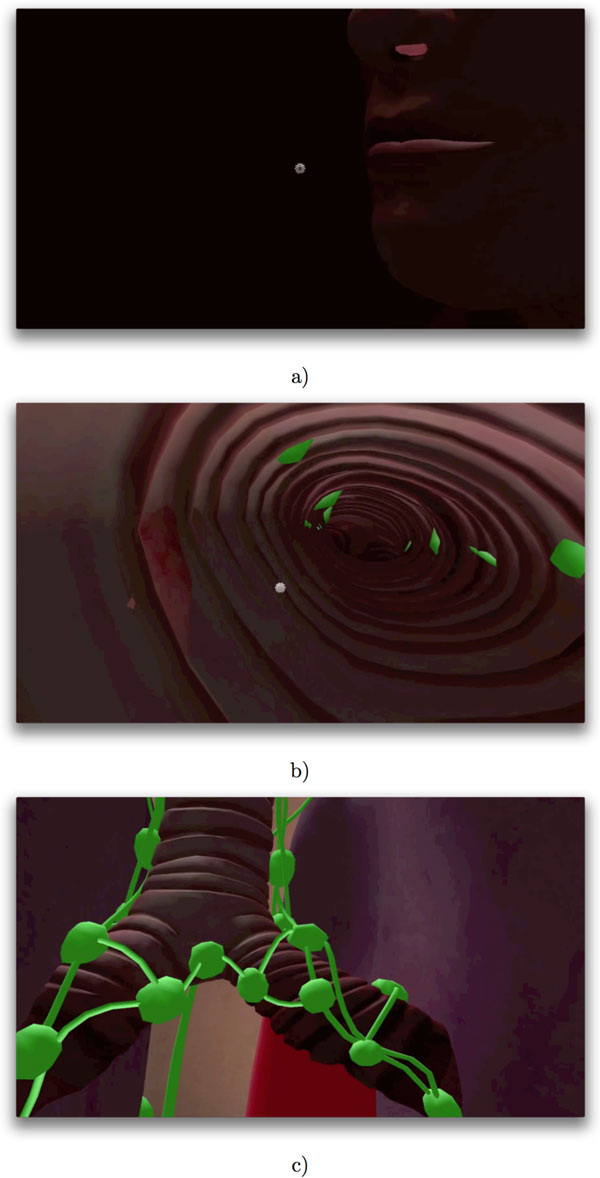
**Virus entry**. The computer model with the airborne Influenza A virus in the context of the LINDSAY Virtual Human model. This diagram illustrates the entry of the virus via the nasal cavity (a), into the upper respiratory tract of a host (b). The green shapes represent lymph nodes in the vicinity of the upper respiratory tract (c).

Influenza A is a member of the *Orthomyxoviridae *family of viruses which affect humans, other mammals and some species of birds. The virus is generally transmitted from infected individuals through the air via coughs or sneezes and infects the epithelial cells lining the lung and throat. The Influenza A virus multiplies by utilizing the host cell's own replication and protein synthesis machinery to produce copies of the virus, which spread out to infect more tissue [18]. Figure 1 illustrates the airborne Influenza a virus in the context of our virtual human model, which is the basis for our simulation.

### Modeling and simulating the human immune system

Natural immune system processes have been inspirational for the modeling of adaptive and learning systems in the field of artificial intelligence [19,20]. We do not consider these machine learning aspects here. Mathematical models, based on differential equations, have been used to simulate changes of concentrations of immune system agents and large-scale regulatory and defensive actions of the immune system [11,12,21]. Lee et al. [22] provide a comprehensive mathematical model of the specifics of Influenza A Infection. They also divide their system into compartments: the lung and the lymphatic system. Communication between compartments is implicit in their mathematical model as there is no difference in scale or any concrete notion of location and distance. While mathematical models do provide accurate results and some conceptual understanding of the overall dynamics, they lack interactive visualizations and are not easily expandable for larger, more integrative systems.

Agent-based modeling, in the form of cellular automata has been used to simulate the processes of the immune system [23]. This work is extended in [24] by introducing cell-mediated interactions to a purely humoral immunity model.

Jacob et al. [10] present an immune system simulation using the concept of virtual swarms, where simple, yet large numbers of particles interact in 3-dimensional scenarios. This work is built upon in [13], where different compartments across an abstracted human body are introduced. Similar to our work, simple agent rules are used to create complex interaction networks across different compartments connected through a data exchange channel. What we have added in the simulations we discuss in this paper are (1) more advanced visualizations, (2) physics-based models that drive agent interactions, and (3) running our immune system models within a 3-D virtual anatomy model of a human male.

For programming the behaviours and properties of agents we use an object-oriented architecture. In this approach, objects can have internal information (such as states) and are able to send and receive certain messages. Furthermore, the inheritance model is of high importance as it allows common behaviors, such as cell motility, to be implemented at a higher level and shared by the sub-class implementations of the different types of individual cells. Bersini provides some insight of how to use object-oriented programming to build the different agents of the human immune system [25].

Integrating different models across many levels of scale is beginning to eliminate what most researchers consider the "bottom-up" approach, where systems are built from atomic parts. In the "bottom-up" approach systems become problematic when attempting to model a realistic process, because of the extremely large number of entities that simultaneously interact. As suggested by Noble [26], building such multi-scale models "middle-out" seems to be a more feasible approach. By creating a connected multi-scale system, changes in a process (such as an infection) can ripple across the different levels of abstraction, to inform the whole system efficiently. The Multiscale Systems Immunology (MSI) project attempts to model the immune response over many different levels of temporal and spatial scales [27] and has been inspirational for our multi-scale models.

## Methods

**Figure 2:**
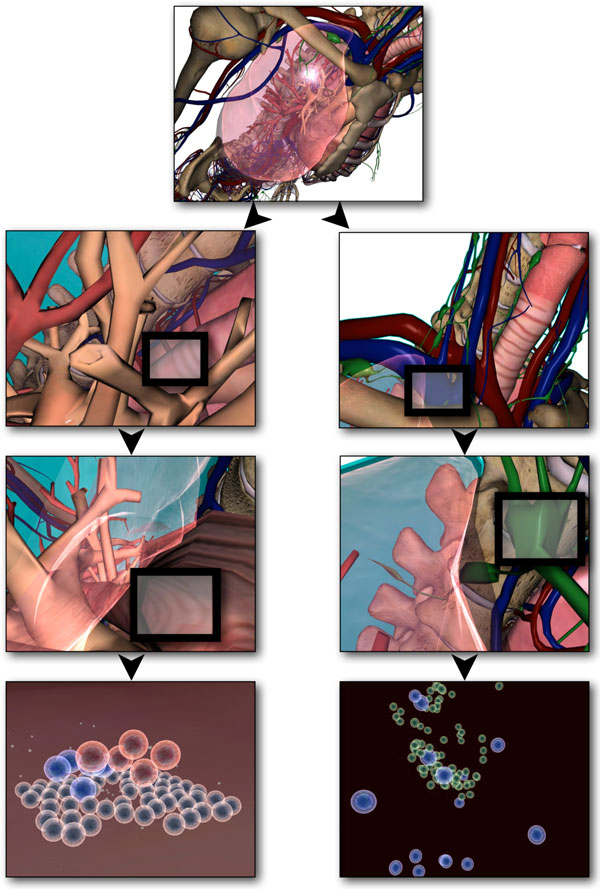
**Anatomical context of the simulation within the LINDSAY composer**. Within the LINDSAY simulation environment, one can represent different simulations within their correct anatomical contexts. This figure depicts the views as a result of interactive zooming from a high level of abstraction to a lower, more fine grained scale. In the multi-scale LINDSAY Virtual Human environment, different simulations can be represented within the anatomical context of interest.

As part of the *LINDSAY Virtual Human *project [28], we are developing a 3-dimensional, interactive computer model of male and female anatomy and physiology to be used for medical education. One key characteristic of *LINDSAY *is the integration of computational models across scales in order to simulate physiological processes from the body level to the level of organs, tissues, cells, and sub-cellular structures. This is similar to the *Physiome *project [29,30], yet our main focus is on medical education with a high degree of interactivity for our simulations, rendered in 3-D, and live navigation across scales (Figure 2).

### LINDSAY composer modeling environment

*LINDSAY Composer *(LC) [31] is a software suite for creating hierarchical 3-D models and visualizations. The modeling environment consists of a collection of component engines for physics, graphics, cameras, user interaction, and agent behaviour rules. Each engine is responsible for controlling the execution of its respective components at each step of the simulation. Component engines can be split into two groups: native engines and external engines. Native engines, such as the physics and graphics engines, are always present within the LC framework. In the model discussed here, the Immune System simulation is built as a plug-in to *LINDSAY Composer *with its own engine loaded at start-up.

**Figure 3:**
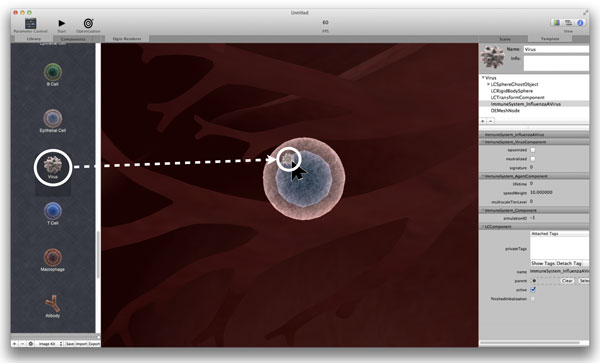
**Drag-and-drop interface**. Using the simple drag-and-drop interface of the LC to infect a cell with a virus particle from the component library (left). The dragging feature is implemented by ray-tracing in 3D space to detect surfaces directly underneath the mouse cursor.

The Lindsay Composer allows a user to construct and add objects to a simulation using a static component library. The component library contains templates of possible agents and other controllers which can interact with the current simulation. Figure 3 illustrates how one can drag a virus particle from the component library onto an epithelial cell to cause infection.

**Figure 4:**
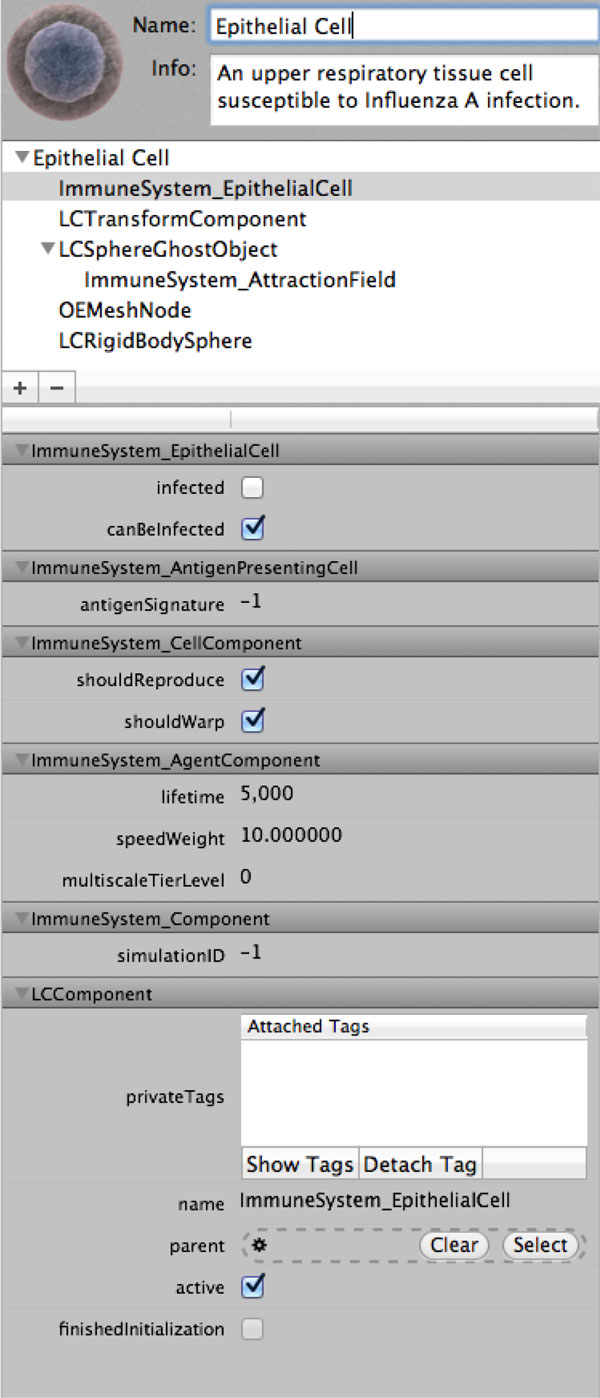
**Component view of an agent**. Illustration of the component hierarchy for an agent. The bottom hierarchy represents the object inheritance map of the "ImmuneSystem EpithelialCell" component, with its own attributes aside from those inherited from super components.

Entities simulated by the LC are represented as hierarchies of components that define their respective states and behaviors. For example, Figure 4 illustrates the component hierarchy for an epithelial cell, in diagrammatic representation and how this organizational structure is accessible through the LC user interface. In the component hierarchy, we see that an epithelial cell component consists of five child components, of which one is nested:

- *ImmuneSystem_EpithelialCell *defines the state and interaction rules of the epithelial cell agent.

- *LCTransformComponent *defines the location, size and orientation of the agent in 3-dimensional space.

- *LCSphereGhostObject *allows the agent to be aware of other physics objects inside the radius of the ghost object, which is used to create a virtual awareness space around an object. This object contains a child--the *ImmuneSystem_AttractionField *component-- which attracts certain other agents (such as viruses) towards the current epithelial cell.

- *OEMeshNode *gives the agent a graphical representation, which contains a 3-dimensional mesh, textures, colours, shadows and animations.

- *LCRigidBodySphere *contains all of the physics related information such as mass, acceleration and collision shape.

The human immune system encompasses processes across a wide range of scale: starting with the organismal level, one can integrate interaction processes at the organ level (e.g., inside the thymus), at the tissue level (e.g., inside lymph nodes), at the intra-cellular interaction level (e.g., between bacteria and macrophages) and, finally, at the inner-cell processes such as protein synthesis and gene regulation. The LC framework allows the developer to integrate different modeling techniques across multiple scales (Figure 2) into one comprehensive model. In the case of the immune system, the simulations at a higher level of scale would include the bone marrow, thymus, lymphatic system and circulatory system, while the simulation at a lower scale would represent individual lymph nodes, site of infection and the process of inflammation.

### Agent-based model of the human immune system

**Figure 5:**
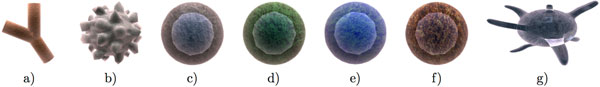
**Immune system agents**. The types of agents in our model (here not depicted to scale): antibody (a), virus (b), epithelial cell (c), B cell (d), T cell (e), macrophage (f) and dendridic cell (g).

**Figure 6:**
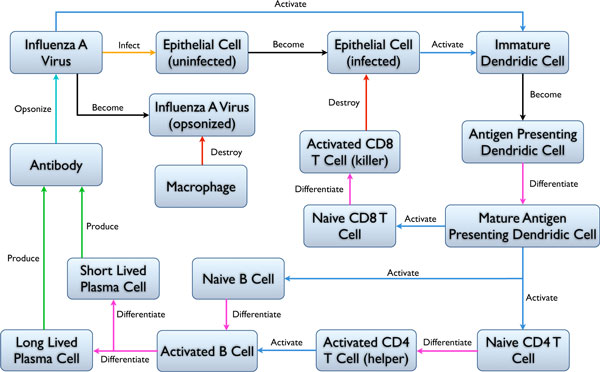
**Agent interaction rules**. Diagram of agent interactions of our immune system model. Coloured arrows represent the different types of interactions between agents.

The agents in our current immune system model are: epithelial cells, viruses, antibodies, B cells, T cells, macrophages and dendridic cells (Figure 5). The behavior of the agents is dictated by sets of rules as well as their internal states. For example, an epithelial cell can be either infected or not infected. Similarly, a virus may be active or opsonized. The state of each cell changes dependent on what interactions it experiences with the other agents as well as the current state of the environment. The agent interactions we have implemented are illustrated in Figure 6.

**Figure 7:**
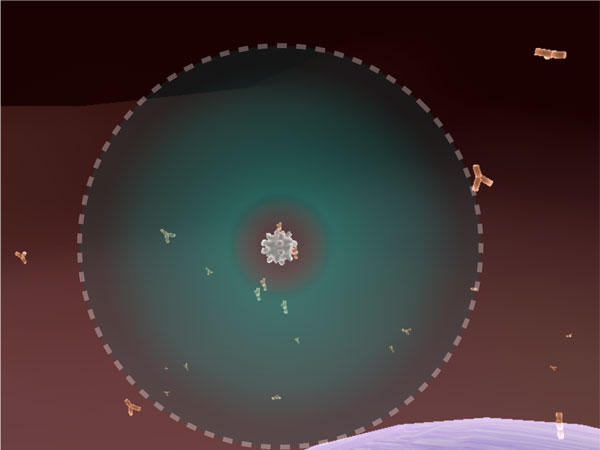
**Agent attraction**. A snapshot of the attraction between a virus particle and antibodies. Upon contact with the antibody, the virus is neutralized. The bounded green area (ghost object) around the virus represents the invisible attraction sphere.

Due to current computational limitations, it is impossible to construct agent-based simulations that represent the exact number of entities found in nature. Therefore, we employed some constraints on our agents to simulate qualitatively correct interactions on a large scale with only a limited number of agents (in our case a few thousand). Since we have such a relatively small number of agents, the chance that two agents will collide randomly is low. Therefore, all our agents have attraction spheres around their actual physical meshes. This allows agents in close proximity to have a higher chance of becoming aware of and interacting with each other. For example, an epithelial cell can attract a virus, and similarly, a virus can attract an antibody (illustrated in Figure 7). T cells, B cells and macrophages also have their respective attraction fields. Once in an attraction field, an agent *α *will break out of the attraction with probability *∅_break_*(*α*) to ensure that overall stochastic motion is preserved. This approach gave us more reproducible results over multiple simulations, rather than basing the physical interactions solely on random motion.

#### Lung tissue

**Figure 8:**
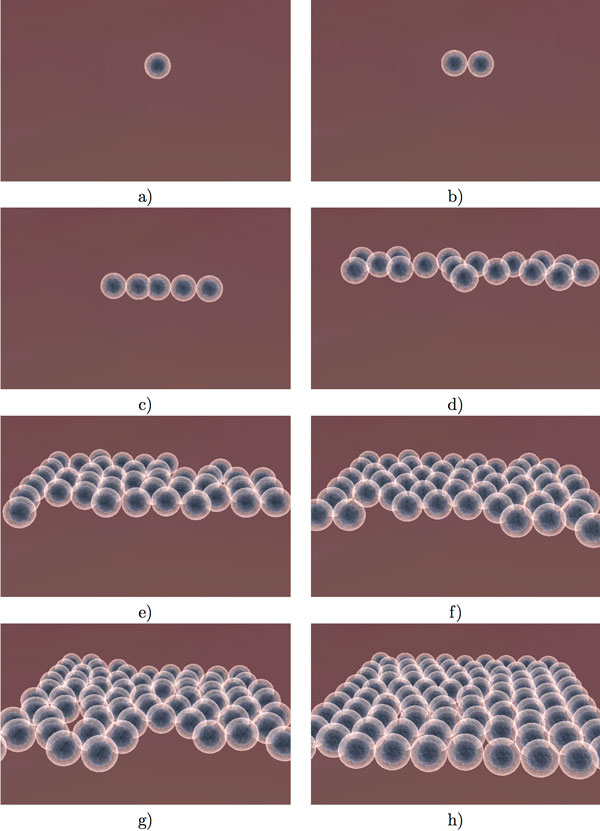
**Epithelial cell division**. Illustration of natural division of epithelial cells. The decision to divide is dependent on the number of neighbours adjacent to each epithelial cell.

The simulated lung tissue consists of approximately 200 epithelial cells. The cells are not statically placed in an aligned fashion, but rather allowed to reproduce and arrange themselves based on physical interactions (Figure 8). The dynamic self-arrangement of the cells allows our simulation to be bound by any arbitrary shape -- such as a vessel -- instead of simply a square arrangement. Replication is triggered if the cell has enough energy and the neighbor count is less than 4 adjacent cells, that is there is still enough space to replicate.

Epithelial cells (∈) are susceptible to be infected by an Influenza A Virus (*v*) with a given probability *p_int_*(∈*,v*) upon direct mesh-to-mesh collision. After infection, the cell lives for another ∈*_incubation _*= 200 iterations, followed by cell death and subsequent release of ∈*_virusesreleased _*= 5 new virus particles. In addition, the lung tissue contains 12 initial immature dendridic cells residing in the tissue. These cells become activated upon contact with viral particles, and are able to migrate to the lymph nodes to activate the B and T lymphocytes.

#### Lymph node

**Figure 9:**
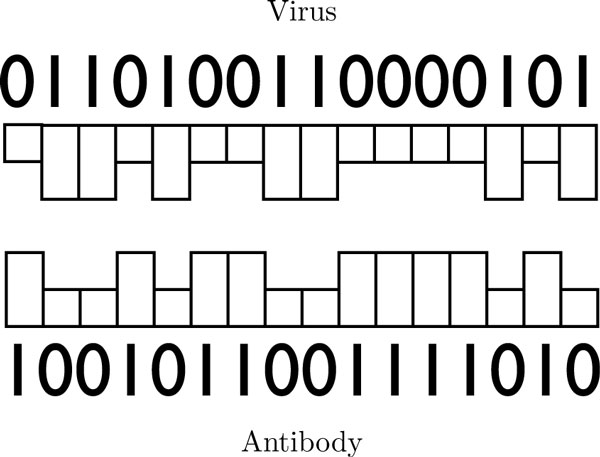
**Antigen and antibody signature**. Illustration of the viral and antibody 16-bit signature. In this figure, the antibody signature perfectly complements the virus signature.

The simulated lymph node is initialized with 15 naive B and 15 naive T cells. When there is no infection detected, the cells maintain a constant population over time. B cells also have a phenotypic signature -- represented in our model as a 16-bit integer -- for immunity against a specific antigen (Figure 9). We define a matching signature which complements the viral signature bit-by-bit (Figure 9). The affinity of an antibody signature to that of a virus is calculated using the *XNOR *binary operator. In the beginning, no B cells have the same signature as the virus, this is acquired through multiple stages of division and clonal selection. Through division, B cells have a random chance to get assigned a new antigenic signature. The B cells that have a high affinity to the viral signature, have a lesser chance to die and higher chance of reproduction. The opposite is true for the B cell that have a low affinity to the viral signature. Those that match he viral signature proceed to divide and release antibodies into the environment. A percentage of the cells that divide from activated B cells become memory B cells and live throughout the entire simulation. The same mechanism for clonal selection is applied to T cells. Some T cells become helper T cells that promote proliferation of B cells and other T cells within the lymph node.

### Simulation distribution

Our experiments look at two different compartments of the human immune system that are able to communicate and coordinate asynchronously. The simulation of the lymph node is executed on a different computing node than the tissue simulation. Our developed protocol allows us to build large networks of simulation nodes working together to form a large-scale distributed simulation system. The network setup as well as the communication between the tissue and the lymph node are controlled through distributed objects (from the Cocoa Objective-C Library), which not only work over a network but also between threads and CPUs, whether on the same computer or not. Therefore, the system is not restricted to one simulation per machine, but rather one simulation per computing core over a large network, which maximizes efficiency with respect to message exchange.

**Figure 10:**
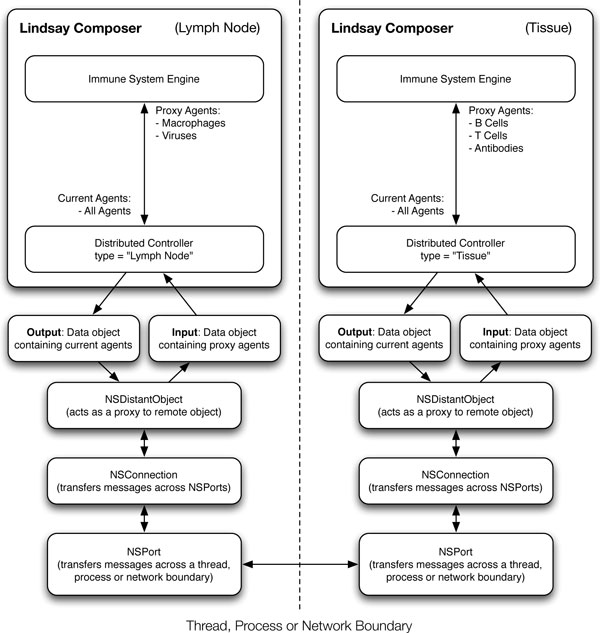
**Information exchange between distributed controllers**. Illustration of the communication between different distributed controllers over a network. In this figure, we show the interaction between a lymph node distributed controller and a tissue distributed controller. This can be extended to include any number of distributed controllers communicating with each other, that can represent other immune system processes at different scales.

In our simulation, the information exchanged between the lymph node and tissue is rather basic. The lymph node distributed controller feeds its agent numbers and properties to other controllers that are connected to it. Since the tissue is connected, it will get informed of changes in numbers of certain agents that it is interested in. For example, the tissue controller is interested in knowing about the number of B cells, T cells and antibodies produced in the lymph node. On the other hand, the lymph node controller is interested in the number of contacts of dendridic cells with viral particles and activated antigen presenting cells in the tissue. At each simulation step, the respective controllers broadcast their current states over the network, allowing asynchronous coordination. Figure 10 illustrates the exchange of information between a lymph node distributed controller and a tissue distributed controller.

The information received by each distributed controller affects the state of the simulation environment or, in this case, the state of the immune system engine of the respective simulation. The agents controlled by the engine are then able to use the new state provided by the engine to inform their decisions, aside from their current physical interactions.

The distributed approach to our system of simulation is highly flexible for extending it over multiple scales. The distributed controllers are not restricted to communicating across simulations of the same type, but can connect data from simulations over multiple scales. For example, one can introduce a differential equation-based model of lymphocyte growth and development in the bone marrow and link it to the agent population in the circulation and in the lymph nodes.

#### Limitations

A limitation of our system lies in different time offsets that can arise from a distributed simulation. Presently, at each distributed controller we make no adjustment with regard to time. That is, if a node is slowing down (e.g., due to limited computing resources) the other nodes connected to it still run at their normal rates. The discrepancy is somewhat accounted for in the fact that the slow node does not receive any updates, and therefore does not broadcast any updates outside of its own rate of execution. This stabilizes the system such that the nodes that require information receive the information more slowly, thus eventually matching the same "simulation" speed as the slowest node. It is worth noting that the "simulation" speed is not the frame rate or execution time, but rather how fast certain events happen inside the context of the simulation.

## Results and discussion

Table 1 summarized all the biological parameters that have been taken into account in our simulation. These parameters have been set in order to reflect the correct biological behaviours as much as possible.

**Table 1:** Simulation Parameters.

	Name	Value
	1 day	200 time steps

Virus	Lifetime	200 time steps (24h)
	incubation time	150 time steps (18h)
	probability of infection	0.1
	number of viruses released	5

B Cell	short lifetime	2500 time steps (10.5d)
	long lifetime	3000000 time steps (>40yr)
	probability of becoming memory	0.2
	number of clones generated	5
	antibodies released	20
	probability of releasing antibodies	0.05

T Cell	short lifetime	2500 time steps (10.5d)
	long lifetime	3000000 time steps (>40yr)
	probability of killing infected cell (CD8)	0.1
	probability of becoming memory	0.2

Dendridic Cell	probability of apoptosis (contact with CD4 T cell)	0.001
	probability of activation (contact with virus)	0.1

Antibody	Lifetime	500 time steps (2.5d)
	probability of neutralization of virus	0.5

Epithelial Cell	probability of reproduction	0.002

The default biological parameters used in our simulations. All probabilities are computed once per time step. In order to set up a realistic time scale, 200 time steps is equivalent to 1 day.

**Figure 11:**
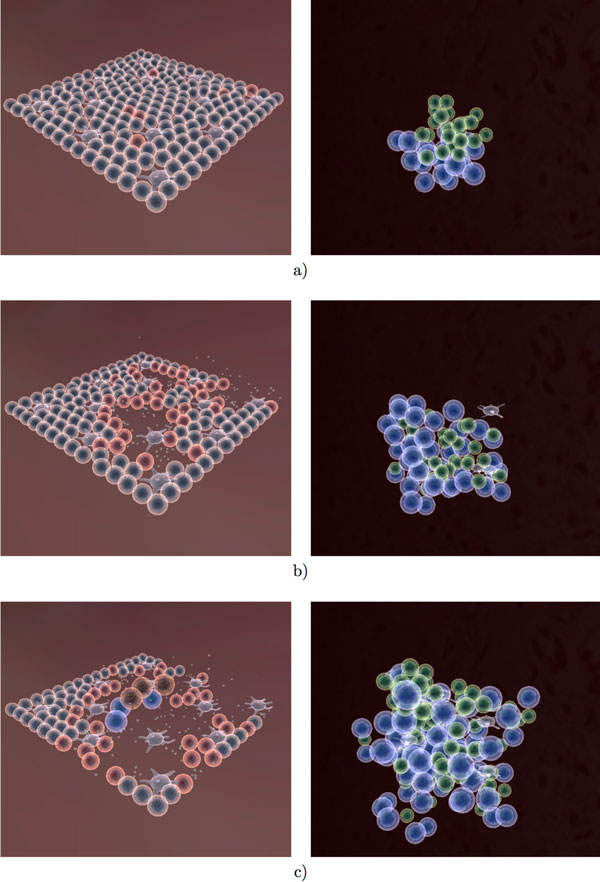
**Visualization of the immune response to influenza A infection (Part 1)**. Agent interactions in both tissue (left) and lymph node (right) as a result of infection with the Influenza A virus. (a) The virus has just infected the tissue cells into the lung, where red cells represent infected cells. At the same time, there is not yet any immune activity in the lymph node. (b,c) As the infection progresses, the immune response can be observed both in the tissue (cell-mediated) and in the lymph node (humoral).

**Figure 12:**
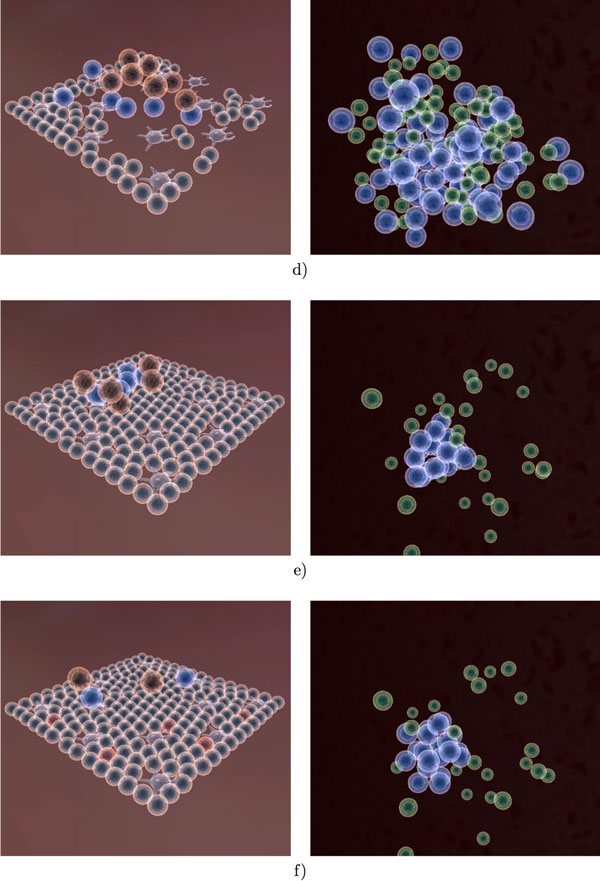
**Visualization of the immune response to influenza A infection (Part 2)**. (d,e) The initial infection is eliminated and the simulation reaches a steady state. (f) We reintroduce the virus into the tissue. Infected epithelial cells are depicted in red.

**Figure 13:**
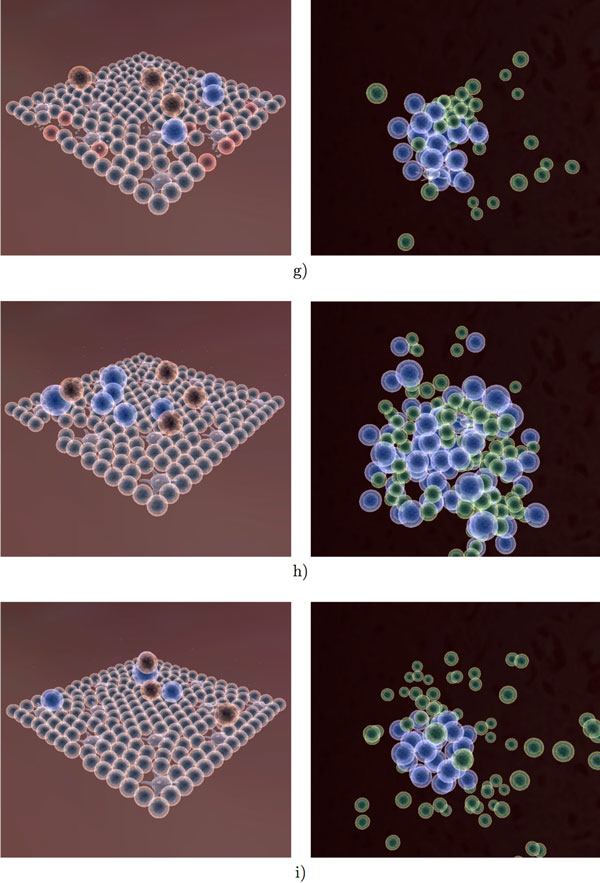
**Visualization of the immune response to influenza A infection (Part 3)**. A large immune reaction to occur in a short amount of time (g,h,i), as a result of reintroducing the virus into the tissue.

Figures 11, 12 and 13 provide an overview of the overall simulation. In Figure 11a figure the virus is introduced to a few cells on the edge of the tissue. The infection then spreads through the tissue and causes significant damage. At the same time, lymphocytes have begun the selection process (Figure 11b). Once the correct signature (encoded as a 16-bit integer) has been found, the B cells and T cells proliferate at a rapid rate. Several cytotoxic T cells can be observed at the site of infection as well as an increased number of macrophages. The antibody produced in the lymph node is transported to the tissue in order to neutralize the virus. Once the infection has been cleared, the tissue regenerates and the lymph node ceases immune activity. Long lived and memory lymphocytes remain in circulation for a long time after the initial infection.

**Figure 14:**
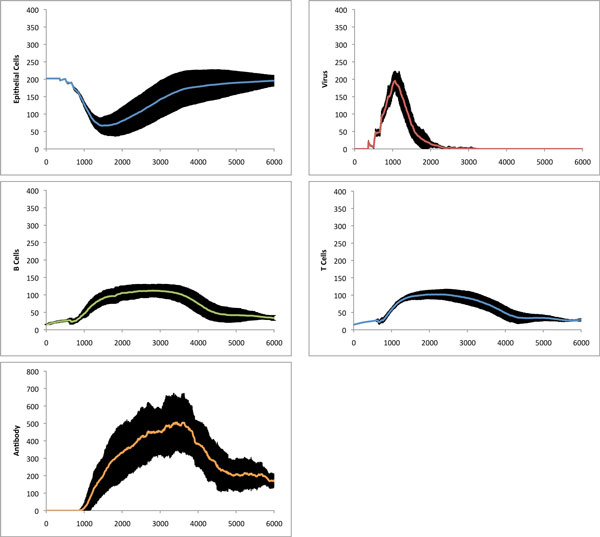
**Overall immune response to influenza A infection**. Influenza A infection: summary of 20 simulation runs with control parameters as described in Table 1. The horizontal axis represents iteration time steps. The vertical axis represents the number of agents. Our simulation runs for 6000 time steps which is equivalent to 30 days.

We performed 20 simulation runs using the parameters in Table 1, with the results illustrated in Figure 14. We included error bars to illustrate the stochastic nature of our simulations. While there is significant variation over the different simulations, the outcome and overall behavior is consistent. None of the simulations resulted in a complete destruction of the tissue nor in hyperactivity of the immune system. We compare our results to a robust and comprehensive mathematical model of the Influenza A infection, described in [22]. Considering the relatively low numbers of agents as well as the stochastic nature of our simulation, our results (Figure 14) agree with those obtained in [22].

**Figure 15:**
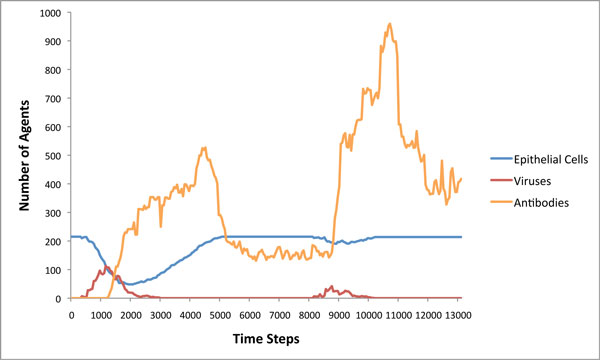
**Adaptive immune response to influenza A infection**. Result of reintroducing the same virus after the initial immune response. It is noticeable that the secondary infection with the same agent is suppressed much more effectively and the duration of the infection is reduced as well.

To test the adaptive capabilities of our immune system model, we re-introduce the same virus into the tissue after the initial infection has been cleared (time step 8000 in one of our experiments in Figure 14). A rapid response from the lymph node can be observed due to the memory lymphocytes (Figure 13h). While the virus still spreads through the tissue during the second exposure, there is not as much observable damage, as the virus is eliminated much faster than before. The overall adaptive response is illustrated in Figure 15, where one can see a clear distinction between the first and second immune response to the Influenza A virus. The overall adaptive behavior of our model is similar to that described in immunology textbooks [32,33]. Not only is the maximum number of viruses less during second exposure, but the damage to the tissue is significantly less than during the first exposure. The cell-mediated reactions start much faster but are not as intense during second exposure as the virus is eliminated more quickly.

## Conclusions and future work

This paper presents our latest work on simulating the decentralized processes of the human immune system within the context of the *LINDSAY Virtual Human*. The *LINDSAY Composer *environment provides a wide array of state-of-the-art simulation techniques including advanced graphical visualization, realistic physical interactions and intuitive live-interaction interfaces. One of the strengths of our immune simulation is the visualization aspect, because it provides dynamic illustrations on the essential concepts of the well-orchestrated system of agent interactions that eventually create complex emergent behaviour such as the acquisition of natural immunity to harmful pathogens. We have created a framework for distributing multiple simulations over a network to increase the efficiency and scalability of our system. Our hope is that by introducing more simulations that work together in a decentralized fashion, we can achieve an advanced and well-connected network of physiological simulations that can enhance our fundamental knowledge of the human body.

In order to produce agent interaction dynamics that are close to (or even the same as) those found in natural biological processes, we are currently applying evolutionary optimization techniques to fine tune the multitude of parameters of our models. At this point, we have gathered some preliminary, yet promising results with other agent-based models [34].

We consider *in silico *experiments and their associated modeling and optimization techniques as essential components in further enhancing our capabilities of simulating a whole-body, decentralized immune system, to be used both for medical education and research as well as for virtual studies in immunoinformatics.

## Competing interests

The authors declare that they have no competing interests.

## Authors' contributions

Vladimir Sarpe (VS) is responsible for building the immune system model. VS was also tasked with designing and evaluating the experiments related to the immune system as well as parameter optimization. Christian Jacob (CJ) has provided supervision, feedback and previous work on simulating immune systems and has contributed to the writing of this paper.

## Declarations

This article has been published as part of *BMC Bioinformatics *Volume 14 Supplement 6, 2013: Selected articles from the 10th International Conference on Artificial Immune Systems (ICARIS). The full contents of the supplement are available online at http://www.biomedcentral.com/bmcbioinformatics/supplements/14/S6.
